# Robotic excision of a retrocaval ancient schwannoma: a case report

**DOI:** 10.1093/jscr/rjae101

**Published:** 2024-03-06

**Authors:** Bianca Marquez, Emmanuel Luciano, Maher Ghanem

**Affiliations:** Department of Surgery, Central Michigan University College of Medicine, Saginaw, MI 48601, United States; Department of Surgery, Central Michigan University College of Medicine, Saginaw, MI 48601, United States; Department of Surgery, Central Michigan University College of Medicine, Saginaw, MI 48601, United States

**Keywords:** case report, robotic surgery, ancient schwannoma, retrocaval schwannoma, ganglioneuroma

## Abstract

We report a case of a robotic-assisted excision of a retrocaval ancient schwannoma. A 40-year-old female presented with generalized weakness and abdominal pain that led to the diagnosis of a retroperitoneal mass adjacent to the pancreas and inferior vena cava. Because of the clinical, imaging, and needle biopsy findings, the patient underwent an elective robotic-assisted retroperitoneal exploration. We provide an overview of the pathology and highlight the significance of utilizing a minimally invasive approach for excision of retroperitoneal masses.

## Introduction

Peripheral schwannomas are the most common benign tumor of the peripheral nerves. Histologically they are categorized by a typical biphasic architecture of Antoni A (cellular with Verocay bodies) and Antoni B (myxoid stroma) fibers. In contrast, an ancient schwannoma is defined as a peripheral schwannoma that has undergone a degenerative process in which the tumor itself has lost the typical histology of a schwannoma, Antoni A and B fibers. Most commonly, the peripheral schwannomas are found within the head/neck region or the extremities. However, to be found within the retroperitoneum is rare and this location accounts for 0.7%–2.7% of all peripheral schwannomas [1, 2]. The incidence of these rare variants is ~0.8% of all soft tissue tumors [2]. A majority of ancient schwannomas tend to be found incidentally and are mostly found in females between the second and fifth decade of life [3]. In the literature review, only one case has been reported of an Ancient Schwannoma involving the major abdominal vessels [4]. This case report presents the diagnosis and surgical management of the first robotic excision of a retrocaval ancient schwannoma on a 40-year-old female.

## Case report

The patient is a 40-year-old female with no significant past medical history who presented for evaluation of generalized weakness, decreased appetite, and abdominal pain. The abdominal examination was unremarkable. The patient initially underwent an ultrasonography of the abdomen that was suspicious for a pancreatic mass.

**Figure 1:**
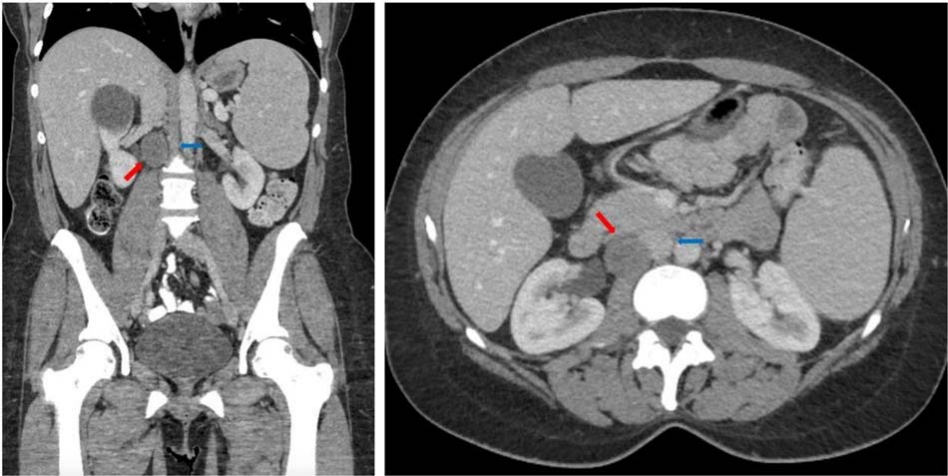
Coronal (left) and axial (right) CT of the abdomen and pelvis showing the retrocaval schwannoma (red arrow) and IVC (blue arrow).

Subsequently, a computed tomography (CT) of the abdomen and pelvis revealed a hypodense lesion located posterolateral to the inferior vena cava (IVC) measuring 28 × 25 mm (see Fig. 1).

The patient underwent a CT-guided core needle biopsy that was consistent with a Schwannoma. The diagnosis of a peripheral nerve sheath tumor was made based on mostly radiologic and biopsy data. We had an extensive discussion with the patient regarding all the options, including surveillance and surgical excision. The decision to proceed with a minimally invasive robotic excision was chosen.

**Figure 2:**
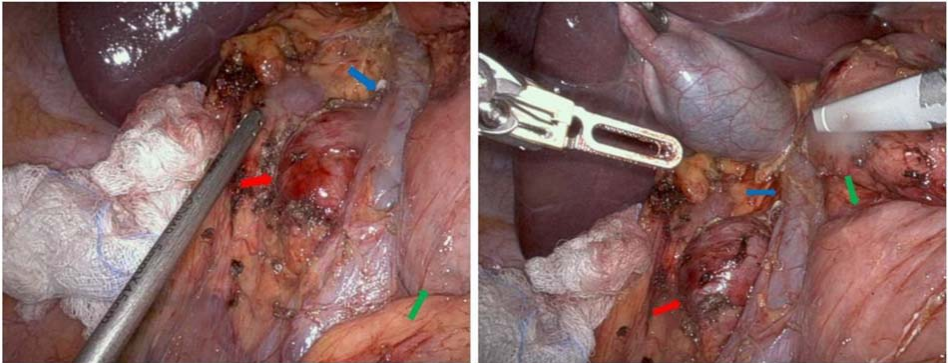
Intraoperative images showing the retrocaval schwannoma (red arrow), IVC (blue arrow), and duodenum (green arrow).

The patient was taken to the operating room and after adequate general anesthesia was induced, pneumoperitoneum was established. Four 8-mm robotic trocars (periumbilical, left upper quadrant, right upper quadrant mid-clavicular line, and right upper quadrant anterior axillary line) and two assistant trocars (12-mm left lower quadrant and 5-mm right lower quadrant) were placed. The daVinci Xi platform was utilized. Cattel-Braasch and Kocher maneuvers were performed to mobilize the right colon and duodenum medially, respectively, in order to expose the IVC and the retrocaval Schwannoma (see Fig. 2).

The right gonadal vessels and right ureter were identified and preserved. A feeding vessel in the superomedial aspect of the mass, originating from the right renal artery, was controlled with hemoclip and divided. The Schwannoma was carefully dissected of the surrounding structures using the robotic vessel sealer energy device. The specimen was retrieved through the 12-mm assistant port. There were no postoperative complications, and the patient was discharged on postoperative Day 1. The procedure was performed at a teaching hospital by the senior hepatobiliary surgery attending.

**Figure 3:**
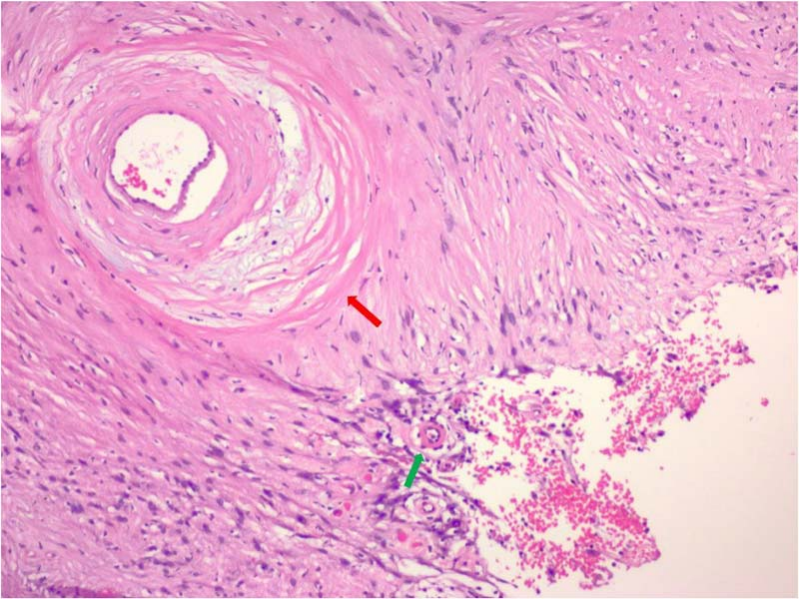
Hematoxylin and eosin stain pathologic slide of ancient schwannoma showing hyalinizing vessel (green arrow) and cystic degeneration (red arrow) characteristic of ancient changes.

**Figure 4:**
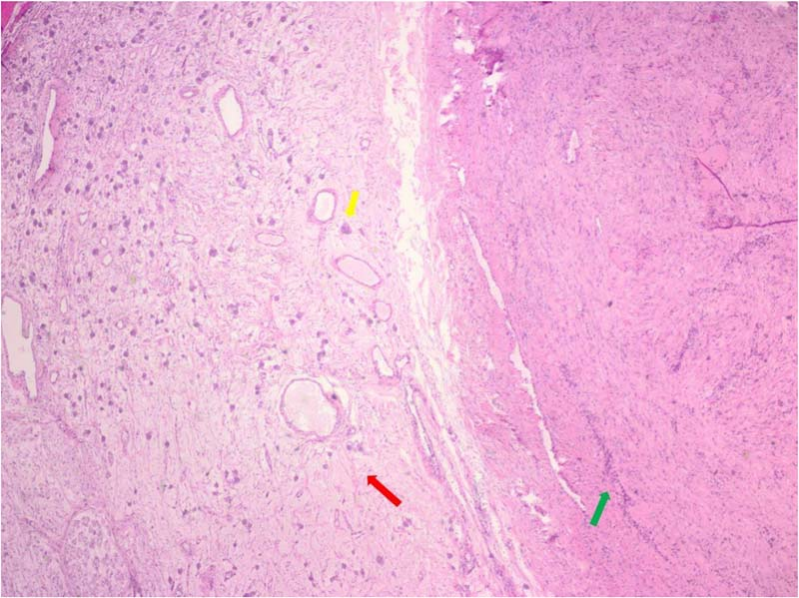
Hematoxylin and eosin stain pathologic slide of ancient schwannoma (green arrow) and adjacent ganglioneuroma (red arrow) with ganglion cell (yellow arrow).

The pathology demonstrated an Ancient Schwannoma (see Fig. 3) with an adjacent ganglioneuroma (see Fig. 4). Because of the benign nature of this tumor, no further workup was required.

## Discussion

An ancient schwannoma is a rare diagnosis and comprises ~0.8% of all benign soft tissue masses [2]. Ackerman and Taylor [5] coined the entity of ancient schwannoma by histological diagnosis. Ancient schwannomas typically depict areas of decreased cellularity (loss of Antoni A), hyalinosis of vessels, calcification, areas of fibrosis, stromal edema, hyperchromasia and may have cystic necrosis with evidence of hematoma formation [6]. Its degenerative qualities are primarily attributed to the growth and aging of the tumor which make it “ancient.” The pathology of the tumor arises from the growth phase of the tumor. Prior studies have correlated that as the tumor increases in size, blood flow decreases leading to vascular insufficiency within the tissue and thus degeneration [7, 8].

Ganglioneuromas are defined as rare, benign neural crest cell tumors. These tumors are largely found along the distribution of the sympathetic chain. They are characterized as a mixture of ganglion and Schwann cells. However, the retroperitoneal location is relatively rare and more commonly found in the pediatric population. The majority of ganglioneuromas reported so far have been in the adrenal gland and the kidney [9].

There is only one case report in the literature identifying an ancient schwannoma within the retroperitoneum. Harouachi *et al*. [4] described an open approach for removal of a retroperitoneal schwannoma within the antero-caval location (anterior to the vena cava). Throughout the literature, there are a few cases utilizing a robotic-assisted approach to resect retroperitoneal masses. As such, Bindel *et al*. [10] described the safety of a robotic approach for the resection of a retroperitoneal schwannoma. Although the mass described in our case was initially biopsied as schwannoma, the pathological analysis of the surgical specimen characterized the schwannoma as the very rare variant of an ancient schwannoma with an adjacent, incidental, ganglioneuroma. The robotic-assisted approach allows for a full 3D detailed view of the operating field with the additive feature of enhanced range of motion through Endowrist technology, facilitating minimally invasive approaches to retroperitoneal masses. To our knowledge, this is the first case of a robotic-assisted excision of a retrocaval ancient schwannoma combined with a ganglioneuroma. We believe that robotic-assisted minimally invasive surgery is an adequate approach for lesions within challenging and complex regions of the abdominal cavity.

Surgeons should be aware of the possibilities of an ancient schwannoma within the retrocaval region and to consider the preoperative steps to achieving a successful complete resection [11].
